# An emergency clinical pathway for stroke patients – results of a cluster randomised trial (isrctn41456865)

**DOI:** 10.1186/1472-6963-9-14

**Published:** 2009-01-21

**Authors:** Assunta De Luca, Danilo Toni, Laura Lauria, Maria Luisa Sacchetti, Paolo Giorgi Rossi, Marica Ferri, Emanuele Puca, Massimiliano Prencipe, Gabriella Guasticchi, the "IMPLementazione percorso clinico assistenziale ICtus Acuto (IMPLICA) study group"

**Affiliations:** 1 Public Health Agency of the Latium Region, Rome, Italy; 2 Department of Neurological Sciences, "La Sapienza" University of Rome, Rome, Italy

## Abstract

**Background:**

Emergency Clinical Pathways (ECP) for stroke have never been tested in randomized controlled trials (RCTs).

**Objective:**

To evaluate the effectiveness of an ECP for stroke patients in Latium (Italy) emergency system.

**Methods:**

cluster-RCT designed to compare stroke patient referrals by Emergency Medical Service (EMS) and Emergency Room (ER) health professionals trained in the ECP, with those of non-trained EMS and ER controls. Primary outcome measure was the proportion of eligible (aged ≤ 80 and symptom onset ≤ 6 hours) stroke patients referred to a stroke unit (SU). Intention to treat (ITT) and per-protocol (PP) analyses were performed, and risk ratios (RR) adjusted by age, gender and area, were calculated.

**Results:**

2656 patients in the intervention arm and 2239 in the control arm required assistance; 78.3% of the former and 80.6% of the latter were admitted to hospitals, and respectively 74.8% and 78.3% were confirmed strokes. Of the eligible confirmed strokes, 106/434 (24.4%) in the intervention arm and 43/328 (13.1%) in the control arm were referred to the SU in the ITT analysis (RR = 2.01; 95% CI: 0.79–4.00), and respectively 105/243 (43.2%) and 43/311 (13.8%) in the PP analysis (RR = 3.21; 95%CI: 1.62–4.98). Of patients suitable for i.v. thrombolysis, 15/175 (8.6%) in the intervention arm and 2/115 (1.7%) in the control arm received thrombolysis (p = 0.02) in the ITT analysis, and respectively 15/99 (15.1%) and 2/107 (1.9%)(p = 0.001) in the PP analysis.

**Conclusion:**

Our data suggest potenti efficiency and feasibility of an ECP. The integration of EMS and ERs with SU networks for organised acute stroke care is feasible and may ameliorate the quality of care for stroke patients.

**Trial registration:**

Current Controlled Trials (ISRCTN41456865).

## Background

Stroke has great impact on social and health systems due to its high incidence, mortality, and residual disability of survivors.

Early identification of symptoms and timely and efficient admittance to stroke units are key elements to improve stroke patient management [1-6]. The implementation of an evidence-based acute stroke care pathway for Emergency Medical System personnel, associated with continuing medical education programs may be one method to achieve this goal [7-13].

### Importance

However, only a few observational comparative and/or registry studies[14] are available to support the role of acute stroke care pathways in the pre-hospital setting, and at present no randomized controlled trials (RCT) have been conducted. A systematic review identified studies based on 'in-hospital' patients, but the extrapolation of the results to a pre-hospital setting is not appropriate[15].

### Goals

We designed a cluster-RCT (c-RCT) (Current Controlled Trials Register Number: ISRCTN41456865)[16] to assess the impact of an emergency clinical pathway (ECP) on the management of acute stroke patients. In this paper we report the final results in terms of proportion of stroke patients appropriately referred to SU and of proportion of patients receiving thrombolytic treatment and differences in organisational times.

## Methods

### Setting

Lazio is a region of about 5,3 million inhabitants, located in Central Italy, that include Rome (3 million inhabitants).

### Selection of patients and randomization

The protocol and methodology of the c-RCT study have been described widely elsewhere[17].

All the Emergency Rooms (ERs) and Emergency Medical Services (EMSs) involved in the study refer patients to two SUs located in urban Rome. The entities (ER and EMS) were located in the center of Rome, in the suburbs and in two districts in the Lazio Region (Viterbo and Frosinone). Participants included all employees at the facilities involved in the study (including ambulance drivers). A total of 47 entities, including 18 ERs and 29 EMS stations (52 ambulances) were eligible for the study.

In the province of Frosinone there were 14 EMS stations and 4 ERs which were working systematically sharing personnel and/or referring patients and for this reasons it was decided to consider them as two large entities (one comprehending the 14 EMS stations and the other comprehending the 4 ERs) so that the entities enclosed in the study were eventually reduced to 30. The 30 entities so created were then randomised in 20 clusters.

### Description of intervention

Clusters were attributed sequential numbers and sample function of STATA 7 (Stata Corp LP 2005) was used to generate random numbers. We utilized the Italian lottery number extracted on the 6th of November 2004 as seed numbers for generating the random sequences. The process was overviewed by an independent statistician.

The EMS and ER health professionals (physicians, nurses and drivers) in the intervention group were trained to apply the ECP procedures using the educational method in line with the experiential learning tradition, described elsewhere[18]. It was organised in three successive phases: 1) interviews with health professionals to identify their learning needs; 2) training the ECP coordinators/facilitators in a residential setting; and 3) on-site training in small groups of health professionals (6–8), led by a coordinator/facilitator. The training was focused on teaching the personnel to identify stroke symptoms, by using the Cincinnati pre-hospital Stroke Scale (CSS)[19] for EMS staff or the National Institute of Health Stroke Scale (NIH-SS)[20] for ER staff; to register the time of symptom onset and age, and consider them as main inclusion/exclusion criteria for thrombolysis; to refer patients with suspected acute stroke to the Emergency Department Stroke Unit when appropriate.

The health professionals in the control group were not aware of being part of the study, did not receive any training and went on managing suspect stroke patients as usual, without adopting specific protocols or standardized procedures and transporting them to the nearest ER.

Outcome data were gathered from EMS run sheets, computerised ER medical records, the Stroke Surveillance System and the Hospital Information System (HIS). In addition, a neurologist at the SUs collected information related to patient treatment. EMS run sheet data were recorded on paper and subsequently entered electronically using Access and Excel programs. All data sets were linked using SAS[21] and Oracle programs[22].

### Methods of measurements and Outcomes

The primary outcome measure was the proportion of eligible acute stroke patients referred to the SU. Based on ECP recommendations, patients aged 80 or less with a focal neurological deficit less than 6 hours after onset were considered eligible for SU referral (eligible stroke patients). To include only true strokes, we considered patients with ICD9CM codes 430–438 reported in the HIS.

As secondary outcome measures we considered: the proportion of strokes diagnosed by EMS/ER and confirmed by the hospital discharge data of the HIS (ICD9CM codes 430–438); the proportion of ischemic strokes "eligible" for SU referral (ICD9CM codes 434 and 436) and suitable for thrombolysis, according to age and stroke onset, that received thrombolysis; the organizational times: for the EMS group – time from dispatch to hospital arrival; for the ER group – time spent in the Emergency Room for the first clinical assessment (first aid ER).

Statistical analyses were performed using STATA8[23]. For the primary and the first two secondary outcomes, we performed both an Intention To Treat (ITT) analysis and a Per-Protocol (PP) analysis on the facilities that completed the study. With the aim of verifying the robustness of the ITT and PP results, the primary outcome was also evaluated on a "metropolitan area" subgroup, including the centre of Rome and suburbs. Organizational times in the metropolitan area were also analysed.

### Data analysis

This is a cluster randomised controlled trial in which the unit of randomisation was the whole personnel of a ER/ES service (cluster). We assumed that cluster level variables may influence the observations. If observations in a cluster are correlated, standard approaches tend to bias p-values downwards and to produce narrower confidence intervals. A basic method is to perform the analysis at patient level correcting coefficients standard errors to account for the cluster design[24]. We used logistic regressions of survey data to obtain odds ratios adjusted by patient age classes (<= 60, 61–75, 76–80), gender, and by geographical area of treatment (city of Rome vs other provinces) with 95% CI corrected for clustering by "survey estimator" procedures[23].

We also reported the design effect (deff) that is the ratio between the variance obtained under our cluster randomised trial design and the variance that we had obtained if had used a simple random sampling.

Considering that some clusters are composed by a couple of ES and ER services, we used the services as the clustering variable in the models.

Estimated Odds Ratios were used to approximate adjusted risk ratios (RRadj) to better represent the true relative risks of eligible patients being referred to the SU, according to the method proposed by Zhang and Yu. [25] Chi square tests were used to compare demographic characteristics of subjects by study arm. Fisher's exact tests were used to compare the proportion of patients treated in the two arms. Given the differences in EMS and ER logistical organization, analytical methods were also performed separately for EMS and ER groups.

### Sample size

In the pre study period, the mean number of suspected strokes transported by the EMS per cluster was about 50 per year and the percentage of correctly transferred patients was 47% (EMS Information System). We assumed an intra cluster correlation of ICC = 0.05. Under this assumption we determined that 25 patients per cluster would obtain a power of 95% to detect a difference of 50% in rates between the two groups, i.e. reaching about 70% of correctly transferred patients in the treated group, with alpha = 0.05.

Similarly, the mean number of strokes treated by the ER per cluster was about 110 per year and the percentage of correctly transferred patients was 14% (Information System of ER). We assumed an intra cluster correlation of ICC = 0.05. Under these assumptions, 55 patients per cluster, should provide a power of 95% to detect a difference of 150% in rates between the two groups, i.e. reaching about 35% of correctly transferred patients in the treated group, with alpha = 0.05. According to these estimates we fixed the duration of recruitment period in 6 months.

### Ethics

The study was submitted and approved by the Ethics Committee of the Regional Agency for Public Health. Informed consent by individual patients was not required, since objects of the investigation were organisational pathways of the Health Services[17].

### Stopping rules

As reported in the study protocol[17] we strictly monitored the study in aim to detect any unexpected events during transportation and troubles deriving from the unavailability of ambulances.

The Agency for Public Health of the Lazio Region is the governmental agency responsible for the collection of emergency visit records, hospital discharge records and mortality records. All analyses were performed on anonymous individual records, while the record linkage was performed by the person with authorized access to personal data and management of the Health databases.

## Results

### Randomisation procedures and cohorts of enrolled patients

**Figure 1:**
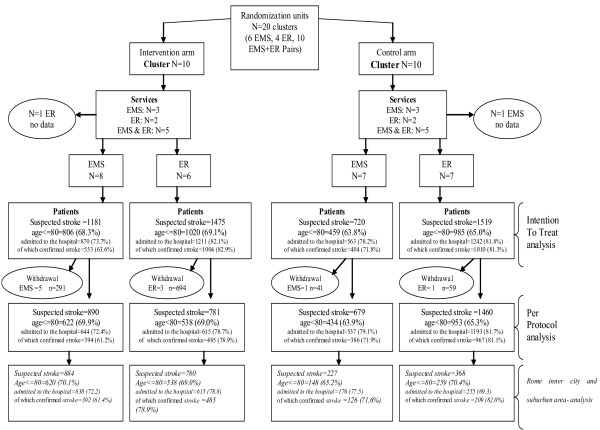
**Flowchart of randomisation procedures and cohorts of enrolled patients**. The figure describes the entities' randomization results, with the description of cohorts of patients involved in the trial.

Figure 1 shows the entities' randomization results, with the description of cohorts of patients involved in the trial and used for the ITT and PP analyses, and for the metropolitan area subgroup analysis. From 30 entities recruited we created 20 clusters of which 10 were pairs of one EMS unit and one ER based on proximity (usually the EMS is located in the same place as the ER), 6 EMS and 4 ERs. Ten clusters were allocated to the intervention arm, with a total of 324 health professionals trained to use the ECP, and ten to the control arm. No patients were recruited by two different facilities in separate study arms. Over a six-month period, 4895 suspected stroke patients who required assistance were involved in the study, 2656 in the intervention arm and 2239 in the control arm. The province of Viterbo did not complete the study, meaning that 10 entities with 1086 patients withdrew, 985 from the intervention and 100 from the control arm, and no patients from Viterbo entities were referred to the related SU.

Table 1 shows the percentage distributions of recruited suspected stroke patients for EMS and ER groups by intervention arm, sex and age class. No statistical differences were found between intervention and control arm in the two groups except for the sex distribution in the ER group (p = 0.03).

**Table 1:** Percentage distributions of patients for EMS/ER groups by intervention gender arm and age class

		**n. suspected stroke Pts**	**% Gender**		***X***^**2**^ **Tests** **P**	**% Age class**				***X***^**2**^ **Tests** **P**
		**n**	**M**	**F**		**<= 60**	**61–75**	**76–80**	**>80**	

**EMS**	I°	1181	44.6	55.4	0.45	19.1	23.4	25.3	32.2	0.06
			
	C°	720	42.8	57.2		14.9	25.0	23.9	36.3	

**ER**	I	1475	51.2	48.8	0.03	12.5	34.6	21.8	31.0	0.14
			
	C	1519	47.3	52.7		11.5	32.6	21.0	35.0	

° I = intervention arm; C = control arm

The withdrawn patients of Viterbo were also compared with those remaining in the study for sex and age, showing higher proportions of males and of older subjects (data not reported).

### ITT analysis

Figure 1 shows the suspected stroke, suspected stroke admitted to the hospital and confirmed stroke admitted to the hospital by arm in the EMS and ERs separately. Overall, from the intervention arm, 78.3% of suspected stroke patients were admitted to the hospital and 80.6% were admitted from the control arm; of them, 74.8% and 78.3% respectively were confirmed strokes (p = 0.2). Among confirmed strokes, 434 (27.9%) patients in the intervention arm and 328 (23.2%) patients in the control arm were eligible for SU referral;106 (24.4%) and 43 (13.1%) of them respectively were referred to the SU (RRadj 2.01; 95%CI 0.79–4.00; deff = 11.3). In particular, the proportion of eligible patients referred to the SU was higher from the EMS intervention arm than from the control arm (RRadj = 4.09,95%CI:1.84–5.59; deff = 4.4), while the proportions were similar in the ER groups (RRadj = 0.92,95%CI:0.23–2.95; deff = 7.6) (table 2).

**Table 2:** Description of eligible stroke patients and Relative Risk of being appropriately referred to the SU by study group

		**Patients with confirmed stroke**	**Eligible** **patients**	**Eligible** **Pts referred to the SU**	**RR of being appropriately referred to the SU**		
		*N*	**N (%)**	**N(%)**	**crude RR**	adjusted RR*	**95%CI accounting for clustering**

**Intention to treat**							

**EMS**	I°	553	109 (19.7)	68(62.4)	3.88	**4.09**	**(1.84, 5.59)**

	C°	404	56 (13.9)	9(16.1)		reference	

**ER**	I	1004	325(32.4)	38 (11.7)	0.93	0.92	(0.23, 2.95)

	C	1010	272 (26.9)	34 (12.5)		reference	

**Tot**	I	1557	434 (27.9)	106(24.4)	1.86	2.01	(0.79, 4.00)

	C	1414	328 (23.2)	43(13.1)		reference	

**Per Protocol**							

**EMS**	I	394	88 (22.3)	67(76.1)	4.40	**4.43**	**(2.26, 5.45)**

	C	386	52 (13.5)	9(17.3)		reference	

**ER**	I	485	155 (32)	38(24.5)	1.87	1.92	(0.78, 3.82)

	C	967	259 (26.8)	34(13.1)		reference	

**Tot**	I	879	243 (27.6)	105(43.2)	3.12	**3.21**	**(1.62, 4.98)**

	C	1353	311 (22.4)	43(13.8)		reference	

*Metropolitan Area*							

**EMS**	I	392	88 (22.4)	67 (76.1)	2.79	**2.99**	**(1.11, 3.59)**

	C	126	22(17.5	6(27.3)		reference	

**ER**	I	485	155 (32)	38(24.5)	0.81	0.58	(0.31, 0.99)

	C	209	53(25.4)	16(30.2)		reference	

**Tot**	I	877	243(27.7)	105(43.2)	1.47	1.86	(0.64, 2.93)

	C	335	75(22.4)	22(29.3)		reference	

In Bold statistically significant adjusted relative risk and 95% confidence interval

*RR adjusted by age, gender and geographical area of treatment (city Rome vs other districts);

° I = intervention arm; C = control arm

There were 175 (40.3%) eligible ischemic stroke patients suitable for thrombolysis according to age and stroke onset in the intervention arm, and 115 (35.1%) in the control arm; of them, 15 patients (8.6%) in the intervention arm and 2 patients (1.7%) in the control arm received the treatment (p = 0.02) (table 3).

**Table 3:** Description of eligible ischemic stroke patients receiving thrombolysis in SU by study group

		**Intention to treat**		
		**Eligible ischemic** **Pts**	**Thrombolised Pts**	**Fisher's exact tests** **P value**

**EMS**	I°	44	8 (18.2)	0.05
	
	C°	22	0 (0)	

**ER**	I	131	7 (5.3)	0.31
	
	C	93	2 (2.1)	

**Tot**	I	175	15 (8.6)	0.02
	
	C	115	2 (1.7)	

		**Per Protocol**		

		**Eligible ischemic Pts**	**Thrombolised Pts**	**Fisher's exact tests** **P value**

**EMS**	I	35	8 (22.9)	0.04
	
	C	20	0 (0)	

**ER**	I	64	7 (10.9)	0.04
	
	C	87	2 (2.3)	

**Tot**	I	99	15 (15.1)	0.001
	
	C	107	2 (1.9)	

° I = intervention arm; C = control arm

### Per Protocol analysis

Overall, in the EMS and ERs there were 75.3% suspected stroke patients admitted to the hospital in the intervention arm and 80.9% in the control arm, and respectively 69.8% and 63.2% had confirmed strokes (p = 0.2) (Figure 1).

Among confirmed strokes, there were 243 (27.7%) eligible patients in the intervention arm and 311 (23.0%) in the control arm; 105 (43.2%) and 43 (13.8%) of them respectively were referred to the SU (RRadj 3.21; 95%CI:1.62–4.98; deff = 9.0). In particular, for EMS-referred patients the RRadj was 4.43 (95%CI:2.26–5.45; deff = 3.2) and for ER patients the RRadj was 1.92 (95%CI:0.78–3.82; deff = 3.4) (table 2).

There were 99 (40.3%) eligible ischemic patients suitable for thrombolysis according to age and stroke onset in the intervention arm, and 107 (35.1%) in the control arm; 15.1% and 1.9% of them respectively received thrombolysis (p = 0.001). The PP proportions of thrombolysis were higher in the intervention arms than in the control arms from both the EMS and the ER, and both the comparison tests were statistically significant (p = 0.04) (table 3).

### Metropolitan area

In the metropolitan area the RRadj of referring eligible patients to the SU was 1.86, (95%CI:0.64–2.93;deff = 4.1). In particular, for EMS-referred patients the RRadj was 2.99 (95%CI:1.11, 3.59; deff = 4.4), while for ER patients the RRadj was 0.58 (95%CI:0.31, 0.99; deff = 2.3) (table 2).

The overall mean EMS transportation time was 31.8 minutes (SD:15.1) in the intervention arm and 35.8 minutes (SD:13.8) in the control arm.

In the suburban district, the mean transportation time in the intervention arm was about 13 minutes longer for patients referred to the SU than for patients who were referred to other nearby hospitals (table 4).

**Table 4:** EMS and ER treatment/travel mean times by study arm and referral hospital Metropolitan area

		**EMS suspected stroke pts: Time from dispatch to hospital (minutes)**						**ER suspect stroke pts trasferred: Time in the first ER (minutes)**	
	
		**EMS: Total metropolitan area**		**EMS:substrata of Rome center**		**EMS:substrata of Rome suburban area **		**ER: Metropolitan Area**	
	
		**N**	**Mean (SD)**	**N**	**Mean (SD)**	**N**	**Mean (SD)**	**N**	**Mean (SD)**
**I**°	***Referral: SU ***	*219*	*29.2 (12.1)*	*190*	*26.7 (8.8)*	*29*	*47.2 (17.2)*	*148*	*180 (162)*

	***Referral: other hosp***.	*665*	*32.6 (15.9)*	*198*	*30.0 (11.4)*	*467*	*33.7 (17.3)*	*17*	*318 (240)*

	**Total**	**884**	**31.8 (15.1)**	**388**	**28.4 (10.3)**	**496**	**34.4 (17.5)**	**165**	**193 (176)**

**C**°	***Referral: SU ***	*7*	*32.2 (9.2)*	*6*	*30.2 (8.8)*	*1*	*42.0(-)*	*56*	*216 (210)*

	***Referral: other hosp***.	*220*	*35.9 (14.0)*	*163*	*30.3 (9.7)*	*57*	*48.5 (14.0)*	*12*	*288 (240)*

	**Total**	**227**	**35.8 (13.8)**	**169**	**30.3 (9.7)**	**58**	**48.4 (13.9)**	**68**	**228 (216)**

° I = intervention arm; C = control arm

The mean time spent in the first ER was lower in the intervention arm than in the control arm: 193 minutes (SD:176) vs 228 (SD:216) minutes in the whole population, and 180 minutes (SD:162) vs 216 minutes (SD:210) in patients transferred to the SU (table 4).

## Discussion

In this cluster RCT on the effectiveness of an emergency clinical pathway in the pre hospital management of stroke, patients eligible for Stroke Unit referral in the intervention arm were more likely to be actually referred to the SU than those in the control arm. We arbitrarily defined eligibility for SU referral based on the age limit of 80 years, i.e. the age limit for i.v. thrombolysis according to the licence released by the European Medicines Agency (EMEA) .

Moreover, we set the time from stroke onset at less than 6 hours, to have a more homogeneous study population and to facilitate very early stabilisation for as many acute stroke patients as possible, regardless of thrombolysis. Obviously, this does not mean that stroke unit referral should be limited only to these patients, since the Stroke Unit Trialists' Collaboration demonstrated that patients may benefit of stroke unit management irrespective of age, gender and stroke severity[26].

In spite of all the resources devoted to education and organization, considering the rate of patients actually treated with thrombolysis expressed as a proportion of all patients with confirmed diagnosis of stroke admitted to hospital, the overall rate is still quite low (1.7% in the intervention group, PP cohort) showing how the implementation of a functionally efficient network for the treatment of stroke patients is still far from being completed, and how much this study was needed.

Conducting this trial necessitated strong organizational and coordination work, due not only to the various types of health services involved in the study, but also to the different regional management, population socio-demographic characteristics and road networks in the different provinces. In fact the long distances from the SU and sometimes the unavailability of ambulances to transport rapidly stroke patient to SU were the reasons why patients from the province of Viterbo, which had originally been included in the study, did not complete the trial. Since the withdrawal from the trial by such a large area, and its related SU, might have had strong statistical implications, we analysed both the ITT and PP cohorts, and the more homogeneous subgroup of Rome metropolitan area.

The global ITT analysis was not statistically significant because the withdrawal of Viterbo meant that approximately 20% of suspected strokes did not complete the trial. This particularly affected the performance of the ER subgroup, in which we did not find any difference in the proportion of eligible stroke patients referred to SU.

On the contrary, in the PP analysis, although still not statistically significant, the number of eligible patients actually referred to the SU by the intervention ER was twice that of the control group. The difference may be explained by two not mutually exclusive phenomena: a) the higher number of withdrawals in the ER intervention arm than in the control arm might have caused a strong contamination in the intervention arm thus masking the effect of the ECP; b) a self-selection of the ERs that remained in the study may indicate their higher overall quality and interest in applying the ECP, hence exaggerating differences with control group.

On the other hand, in the same period of the previous year the SU did not thrombolyse any stroke patient transferred from the ERs of the intervention arm. Hence, although the proportion of eligible patients referred to the SU by the ERs in the two arms were similar, those from the intervention arm were more likely to be correctly selected for thrombolysis[27]. In fact, in addition to age and time from stroke onset, other exclusion criteria for thrombolysis, reported on the summary of product characteristics of actilyse, were communicated during training, and it is possible that trained ER physicians applied them extensively.

There was a significant improvement in eligible patients referrals by the EMS intervention subgroup both in the ITT and the PP analysis, indicating a strong effect of the ECP. This was also confirmed by the fact that thrombolysis was performed only on patients referred by the EMS intervention subgroup, and in none of those referred by the EMS control group. On the other hand, the EMS intervention arm had a lower rate of confirmed strokes than the control arm, suggesting possible over-diagnosing by the trained EMS personnel. In fact, one possible criticism of ECP training is that more patients with symptoms mimicking a stroke might be referred to a stroke unit, thereby increasing health personnel workload and inappropriate use of resources.

The metropolitan area analysis confirmed the robustness of these results. Regarding organizational times, EMS times from dispatch to hospital were only slightly lower[11]. However, due to the need to transport eligible patients to the SU instead of to the closest hospital we expected a higher mean time in the intervention arm, and the reported slight reduction may be an effect of training.

Length of stay in the first aid ER was also lower, particularly for patients subsequently referred to the SU. Keeping organizational times within an acceptable range is crucial for stroke patient management. Although they are not generalizable to other provinces in the region, which may be quite far from the SU, these results indicate the feasibility of the ECP in terms of organizational times, and suggest the need of an organized network of SUs in our region.

Our study has some limitations. The intervention evaluated in this trial was directed to small teams of emergency medicine professionals, and hence the cluster randomisation was the only solution. Despite involving almost all the region, there were only 20 randomisation units available to perform the trial. Strong non homogeneity among clusters and withdrawal of some important participating centres determined contamination in the ITT analysis, thus reducing the power of the study. Another limit is that information on symptom onset was not taken in all patients by the EMS personnel. Moreover, direct access to CT which, according to the protocol, together with time of onset was necessary to define the eligible stroke patients[17], was not available in the whole study population. This is the reason why we decided to identify confirmed strokes with the HIS, ICD9CM 430–438 discharge codes, generally considered reliable for clinical diagnosis[27-29], while the information on symptom onset was taken from the computerized ER medical reports.

## Conclusion

In conclusion, the present study suggests the potential efficacy, efficiency and feasibility of an ECP, indicating that its implementation through a continuing educational program might facilitate more appropriate and homogeneous pre hospital management of acute stroke patients. At the same time, it suggests that further efforts are necessary to teach the accurate recognition of stroke symptoms, a key concept which must be stressed in training programs[7,11,13]. Our experience indicates that integrating EMS systems and ERs for organised acute stroke care, which must include SU networks, is feasible and may ameliorate the quality of care for stroke patients.

## Competing interests

The authors declare that they have no competing interests.

## Authors' contributions

AD had full access to all the data in the study and takes responsibility for the integrity of the data and the accuracy of the data analysis. AD, MF, GG conceived of the study, and participated in its design and coordination. AD LL participated and coordinated the acquisition of data. LL, AD, PG performed the statistical analysis. AD, LL, PG, MF, DT, MLS, EP participated to the interpretation of data and helped to draft the manuscript. AD, LL, DT, PG, MF, MLS, MP, GG participated to critical revision of the manuscript for scientific content and gave administrative, technical, or material support. GG, MP participated to the study supervision. All authors read and approved the final manuscript.

## Pre-publication history

The pre-publication history for this paper can be accessed here:


